# Supplementary material for: Prospective cohort study reveals unexpected aetiologies of livestock abortion in northern Tanzania
Source: Sci Rep. 2022 Jul 8;12:11669. doi: 10.1038/s41598-022-15517-8 (PMC9270399; doi:10.1038/s41598-022-15517-8)
Supplement: Supplementary file 1 — Supplementary Information. [file 41598_2022_15517_MOESM1_ESM.pdf]

Supplementary Materials (S1A-E) for:

Prospective cohort study reveals unexpected aetiologies of livestock abortion in northern Tanzania

Kate M. Thomas*, Tito Kibona, John R. Claxton, William A. de Glanville, Felix Lankester, Nelson Amani, Joram J. Buza, Ryan W. Carter, Gail E. Chapman, John A. Crump, Mark P. Dagleish, Jo E. B. Halliday, Clare M. Hamilton, Elisabeth A. Innes, Frank Katzer, Morag Livingstone, David Longbottom, Caroline Millins, Blandina T. Mmbaga, Victor Mosha, James Nyarobi, Obed M. Nyasebwa, George C. Russell, Paul N. Sanka, George Semango, Nick Wheelhouse, Brian J. Willett, Sarah Cleaveland, and Kathryn J. Allan

S1, A; Table A: Individual-level prevalence (% seropositive) of exposure to a range of abortigenic agents in livestock in northern Tanzania sampled through: (i) cross-sectional surveillance and (ii) abortion cohort study (acute serum samples only). Clopper-Pearson 95% confidence intervals are also shown [ ].

	**Cattle**			**Goats**			**Sheep**		
**Abortigenic agent**	**Cross-sectional**		**Abortion cohort (n = 71)**	**Cross-sectional**		**Abortion cohort (n = 98)**	**Cross-sectional**		**Abortion cohort (n = 43)**
**Bacteria**	**n tested**	**%**	**%**	**n tested**	**%**	**%**	**n tested**	**%**	**%**
Brucella spp.	3627	4.1 [3.5 – 4.8]	5.6 [1.6 – 13.8]	3342	3.7 [3.1 – 4.4]	0 [0 – 3.7]	2628	2.5 [2.0 – 3.2]	0 [0 – 8.2]
Chlamydia abortus	n/a	n/a	n/a	1429	1.3 [0.8 – 2.0]	1.1 [0.0 – 6.0][a]	1017	0.8 [0.57 – 3.1]	2.4 [0.1 – 12.6][b]
Coxiella burnetii	3602	5.2 [4.5 – 6.0]	2.8 [0.3 – 9.8]	3325	27.9 [26.3– 29.4]	37.8 [28.2 – 48.1]	2620	17.4 [15.9 – 18.9]	20.9 [10.0 – 36.0]
Leptospira Hardjo serovars	3527	22.3 [21.0 – 23.8]	8.5 [3.2 – 17.5]	n/a	n/a	n/a	n/a	n/a	n/a
**Protozoa**									

Neospora caninum	3108	12.6 [11.4 – 13.8]	31.0 [20.5 – 43.1]	479	1.7 [0.7 – 3.3]	1.0 [0.0 – 5.6][c]	412	1.5 [0.5 – 3.1]	0 [0 – 8.2]
Toxoplasma gondii	n/a	n/a	18.3 [10.1 – 29.2]	1420	14.9 [13.1 – 16.8]	23.7 [15.7 – 33.4][c]	1009	10.6 [8.8 – 23.7]	7.0 [1.5 – 19.1]
**Viruses**									
Bovine Herpes Virus 1 (BHV-1)	3013	66.2 [64.5 – 67.9]	28.2 [18.1 – 40.1]	n/a	n/a	n/a	n/a	n/a	n/a
Bluetongue virus (BTV)	299	90.3 [86.4 – 93.4]	n/a	186	86.0 [80.2 – 90.7]	n/a	367	68.7 [63.7 – 73.4]	n/a
Bovine viral diarrhoea virus (BVDV)	3015	36.0 [34.3 – 37.8]	49.3 [37.2 – 61.4]	n/a	n/a	n/a	n/a	n/a	n/a
Rift Valley fever virus (RVFV)	3582	4.4 [3.7 – 5.1]	21.1 [12.3 – 32.4]	3304	1.4 [1.0 – 1.8]	2.0 [0.2 – 7.2]	2583	2.6 [2.0 – 3.3]	0 [0 – 8.2]

n/a = not applicable - tests not run; (a) n = 90 for *C. abortus* testing in goats; (b) n = 42 for *C. abortus* testing in sheep; (c) n = 97 for *N. caninum* and *T. gondii* testing in goats

S1, B: Histopathology and immunohistochemistry for abortion cohort study

Objective: To perform histopathologic examination and targeted immunohistochemistry on available placental tissues to support diagnosis of aetiology of abortions from ruminant livestock.

Methods:

Abortion events were identified as outlined in the main text of this article. Following an abortion event, where placental tissue samples were available, sections of cotyledonary tissue was stored in 10% buffered formal saline to preserve tissue structure for histopathology and immunohistochemistry (IHC). Formalin-fixed tissue samples were shipped to University of Glasgow, UK under import licenses TARP(S) 2019/07 and Material Transfer Agreements between Kilimanjaro Christian Medical Centre (KCMC) and University of Glasgow, and between KCMC and Moredun Research Institute (MRI), Edinburgh, Scotland.

Histopathology was performed at the University of Glasgow Veterinary Diagnostic Services (UG VDS) with examination conducted by two veterinary pathologists (CM and GC). Available tissue samples were processed routinely and sections (4 µm) were stained with haematoxylin and eosin (HE). Special stains were applied at the discretion of the examining pathologist following standard methods including Gram stains, Giemsa, and Periodic Acid Schiff (PAS) to diagnose the presence of bacteria, fungi, or other micro-organisms. Finally, Von Kossa stain was performed to differentiate mineral deposits, a normal histologic finding in ruminant placenta (Botta *et al.*, 2019), from other causes of granular deposits (e.g., bacteria) within placental tissue.

Subsequently, placental tissue samples that tested positive for *Coxiella burnetii* by *IS1111a* qPCR on any sample were transported to MRI for IHC. Sections of placental tissue were deparaffinized in xylene prior to rehydration to 95% ethanol. After quenching of endogenous tissue peroxidase activity with 3% hydrogen peroxide (Sigma) in methanol (v/v), the sections were washed in water followed by Tris Buffered Saline (TBS). Non-specific antibody binding was blocked with 25% normal goat serum (NGS, Vector Laboratories, Peterborough, UK) in TBS for 30 min prior to overnight

incubation at 4 °C with primary mouse monoclonal anti-*C. burnetii* antibodies (mAb) (clone 3.13; Squarix GmbH, Marl, Germany), diluted in 1/500 in TBS. Visualisation of bound antibodies was by the EnVision Kit (goat anti-mouse horseradish peroxidase conjugate, DakoCytomation, Ely, UK), as per the manufacturer's instructions, followed by addition of chromogen (AEC, Vector Laboratories, Peterborough, United Kingdom) for 10 min. Slides were then washed with tap water, counterstained with haematoxylin for 1 min, washed in tap water again and mounted in ImmunoHistoMount™ (Sigma-Aldrich Company Ltd., Poole, United Kingdom). Negative control sections were comprised of semi-serial sections of all tissues examined by IHC, with the primary antibodies substituted by isotype-matched mouse IgG antibodies. Sections of known *C. burnetii*-positive bovine placenta from a clinical case of Q-fever abortion were also included as positive controls.

Results:

Fixed placental tissue was available for histopathology from 41 (19.1%) of 215 abortion events recorded by the study. By species, this included 32 (45.1%) of 71 cattle abortion events, eight (8.0%) of 100 goat abortion events and one (2.3%) of 44 sheep abortion events. Of abortion events with placental tissue available for examination, 18 (43.9%) met the study case definitions as defined in Table 3 (main text). *Neospora caninum* was the most commonly diagnosed pathogen in eight (25.0%) of 32 cattle abortion events and one (12.5%) of eight goat events with placental tissue available. In addition, Rift Valley fever virus (RVFV) was diagnosed in six (18.8%) of 32 cattle events, bovine herpesvirus (BHV-1) in two (6.3%) cattle, bovine viral diarrhoea virus (BVDV) in one (3.1%) cow and *C. burnetii* in one (12.5%) goat. Of note, one cow met the case definition for both *N. caninum* and BHV-1.

A summary of histopathology findings is shown in Table S1C. Autolysis, ranging from mild to severe, or poor tissue preservation was observed as a feature in placental samples from 38 (92.7%) of 41 abortion events, which limited histopathological interpretation in some cases. Evidence of placental inflammation (placentitis) was the most common pathological lesion identified and was detected in 14 (34.1%) abortion events, from 13 (40.6%) cattle and one (12.5%) goat. Vasculitis also accompanied the placentitis in eight (25.0%) cattle abortion events. Accumulations of bacteria within placental tissue were a confirmed by Gram stain in 11 (84.6%) of 13 abortion events

where Gram staining was performed. In five (45.4%) of these, the bacteria present were of mixed morphology and were interpreted as most likely to be a result of post-mortem contamination. The significance of other bacteria observed is unclear without further bacteriological or metagenomic investigation.

Positive antibody labelling for *C. burnetii* was detected in the placenta of one goat that met our study case definitions for *C. burnetii*-associated abortion. A further seven animals (five cattle, one goat, one sheep) that were positive for *C. burnetii* by *IS1111a* qPCR, but did not meet our study case definitions were also subjected to IHC but no significant antibody labelling, or pathology indicative of *C. burnetii* infection, was detected in any of these samples.

Table S1, B: Summary of results from histopathologic examination of placental samples collected from aborting ruminant livestock as part of the abortion cohort survey, northern Tanzania.

Animal ID	Species	Tissue preservation	Special stains & results	Coxiella IHC	Morphological Diagnosis	Pathogens detected by PCR			Evidence of seroconversion (paired serology)	Diagnosis based on study case definitions
						Placenta	Vaginal swab	Foetal swab		
SEBI-010	Cattle	Marked autolysis	Gram stain: No significant findings	NA	None	No	*Neospora caninum*	*Neospora caninum*	*Neospora caninum*	*Neospora caninum*
SEBI-011	Cattle	Poor tissue preservation	Gram stain: Moderate to high numbers of gram positive and Gram-negative bacteria including large aggregates of gram negative and separately Gram-positive coccobacilli.<br><br>PAS stain: no significant findings	NA	Placentitis, suppurative, lymphohistiocytic	No	*Neospora caninum*	*Neospora caninum*	*Neospora caninum*	*Neospora caninum*
SEBI-013	Cattle	Moderate autolysis, poor tissue preservation	Gram stain: Multifocally low numbers of Gram-positive bacilli are seen scattered and in rare clusters within the tissue<br><br>PAS stain: Positive indicating presence of glycos-aminoglycans<br><br>Von Kossa: Negative	NA	Placentitis, suppurative	No	No	NA	BHV-1	BHV-1

SEBI-016	Cattle	Some autolysis	NA	NA	None	*Neospora caninum*	No	No	*Neospora caninum*	*Neospora caninum*
SEBI-028	Cattle	Marked to severe autolysis	Gram stain: Moderate to high numbers of gram negative coccibacilli within tissue, and layer of Gram-positive cocci bacilli and few rods adherent to the surface of the tissue.	NA	Placentitis, suppurative and histiocytic, with necrotising vasculitis, and multifocal fibrin thrombi	No	No	No	No	None
SEBI-029	Cattle	Moderate to marked autolysis	NA	NA	None	*Neospora caninum*	No	No	*Neospora caninum*	*Neospora caninum*
SEBI-044	Cattle	Marked autolysis	NA	NA	None	No	No	No	No	None
SEBI-046	Cattle	Moderate to marked autolysis	Gram stain: No significant findings<br><br>PAS stain: No significant findings	No labelling	None	*Coxiella burnetii*(Av Ct 36.0)	No	No	No	None
SEBI-054	Goat	No significant preservation problems reported	Gram stain: Few trophoblasts in the area of necrosis have cytoplasm which stains Gram negative, but no distinct bacteria can be seen<br><br>PAS stain: Low to moderate number of trophoblasts contain moderate amounts of PAS positive finely granular material (1um in size)	Variably small to medium amounts of positive granular labelling present within the cytoplasm of small to moderate numbers of trophoblasts	Placentitis, necrotising, suppurative	*Coxiella burnetii* (Av Ct 12.5)	*Coxiella burnetii* (Av Ct 9.3)	NA	NA	*Coxiella burnetii*

SEBI-055	Cattle	No significant preservation problems reported	Gram stain: Very low scattered numbers of Gram-positive rods are seen within autolytic cotyledons	A very small amount of granular labelling within the cytoplasm of a low number of degenerate cells plus a large amount of very pale and diffuse labelling	None	*Coxiella burnetii* (Av Ct 32.2)<br><br>*Neospora caninum*	*Coxiella burnetii* (Av Ct 30.2)	NA	*Neospora caninum*	*Neospora caninum*
SEBI-067	Goat	Mild to moderate autolysis	NA	NA	None	No	No	No	No	None
SEBI-076	Cattle	Marked autolysis	NA	No labelling	None	*Coxiella burnetii* (Av Ct 36.7)<br><br>RVFV	*Coxiella burnetii* (Av Ct 36.5)<br><br>RVFV	RVFV	NA	RVFV
SEBI-079	Cattle	Moderate to marked autolysis	NA	NA	None	RVFV	No	No	No	RVFV
SEBI-084	Cattle	Moderate to marked autolysis	NA	NA	None	RVFV	No	RVFV	No	RVFV
SEBI-097	Goat	Mild autolysis	NA	NA	None	No	No	NA	No	None

SEBI-099	Cattle	Moderate to marked autolysis	NA	NA	None	RVFV	No	NA	No	RVFV
SEBI-102	Goat	Mild to marked autolysis	NA	NA	None	No	No	NA	NA	None
SEBI-103	Cattle	Moderate to marked autolysis	Gram stain: Moderate to high numbers of Gram-negative coccobacilli/short rods (also stain positive with Giemsa) seen in tissue and in adjacent debris on surface<br><br>PAS stain: No significant findings	NA	Placentitis, lymphohistiocytic, with necrotising vasculitis and high numbers of Gram negative, Giemsa positive bacteria	No	No	NA	No	None
SEBI-110	Cattle	Moderate to marked autolysis	NA	NA	None	RVFV	RVFV	RVFV	No	RVFV
SEBI-114	Cattle	Marked autolysis	NA	NA	None	No	No	NA	No	None
SEBI-115	Cattle	Marked autolysis	NA	No labelling	None	*Coxiella burnetii* (Av Ct 36.7)<br><br>RVFV	RVFV	NA	No	RVFV
SEBI-116	Cattle	Variable preservation with moderate to marked autolysis in some sections and	Gram stain: Low numbers of Gram-negative bacteria seen multifocally (Giemsa negative)<br><br>PAS stain: No significant findings	NA	Placentitis, suppurative, necrotising with necrotising vasculitis and fibrin thrombi.	No	No	No	No	None

		reasonable preservation in other areas								
SEBI-117	Cattle	Variable moderate to severe autolysis	NA	NA	Placentitis, lymphohistiocytic with necrotising vasculitis	No	No	NA	No	None
SEBI-122	Cattle	Marked autolysis	NA	NA	None	No	No	NA	No	None
SEBI-127	Cattle	Marked to severe autolysis	NA	NA	Placentitis, suppurative	No	No	NA	No	None
SEBI-130	Cattle	No significant preservation problems reported	NA	NA	Placentitis, lymphohistiocytic with vasculitis and fibrin thrombi	No	No	No	NA	None
SEBI-133	Sheep	Moderate autolysis	NA	A small number of trophoblasts with small to medium amounts of positive labelling within the cytoplasm	None	No	*Coxiella burnetii* (Av Ct 37.6)	NA	No	None
SEBI-135	Cattle	Moderate to marked autolysis	Gram stain: There are low numbers of gram positive bacteria and few gram negative cocci distributed in surface debris throughout the section. Post mortem contamination suspected.	NA	Placentitis, suppurative	No	No	No	NA	None
SEBI-136	Cattle	Variable preservation	NA	NA	Placentitis, lymphohistiocytic with necrotising vasculitis	No	No	NA	NA	

SEBI-168	Goat	Moderate autolysis	NA	A single cell with diffuse cytoplasmic labelling	None	*Coxiella burnetii* (Av Ct 36.3)	*Neospora caninum*	*Neospora caninum*	*Neospora caninum*	*Neospora caninum*
SEBI-169	Goat	Moderate to marked autolysis	NA	NA	None	No	No	No	No	None
SEBI-172	Cattle	Moderate to marked autolysis	Gram stain: Moderate to high numbers of Gram-positive coccobacilli are present admixed with debris on the surface of cotyledons and near the surface of villi	NA	Placentitis, necrotising, suppurative	No	No	NA	No	None
SEBI-175	Cattle	Moderate to marked autolysis	NA	No labelling	None	*Coxiella burnetii* (Av Ct 37.5)	No	No	No	None
SEBI-184	Cattle	Moderate to marked autolysis	Gram stain: Moderate numbers of Gram-positive and Gram-negative rods seen on the surface debris and fewer within stroma. Post mortem contamination suspected.	NA	Placentitis, necrotising, lymphohistiocytic and suppurative with necrotising vasculitis and fibrin thrombi.	*Neospora caninum*	*Neospora caninum*	NA	BHV-1<br>*Neospora caninum*	BHV-1 and *Neospora caninum*
SEBI-185	Cattle	Mild to moderate autolysis	NA	NA	None	*Neospora caninum*	No	No	*Neospora caninum*	*Neospora caninum*
SEBI-186	Goat	Severe autolysis	NA	NA	None	No	No	No	No	None
SEBI-187	Cattle	Moderate to marked autolysis	Gram stain: Gram-positive and negative bacteria of mixed morphology are present throughout the tissue including within	NA	Placentitis, necrotising, suppurative with vasculitis	No	No	NA	No	None

			blood vessels. Post mortem contamination suspected.							
SEBI-189	Cattle	Moderate to marked autolysis	NA	NA	None	*Neospora caninum*		NA	*Neospora caninum*	*Neospora caninum*
SEBI-190	Cattle	Mild autolysis	NA	NA	None	No	No	Pestivirus (BVDV)	BVDV/BDV	BVDV
SEBI-191	Cattle	Marked to severe autolysis	NA	NA	None	No	No	NA	NA	None
SEBI-202	Goat	Moderate autolysis	NA	NA	None	No	No	No	NA	None

*As defined by study case definitions

Interpretation and conclusions:

***Neospora caninum*-associated abortions:** *N. caninum* was the most commonly diagnosed aetiology of abortion in cattle based on study case definitions but placental histopathology results were mixed. In two cases, placental pathology including placentitis and vasculitis was observed and for the remaining six cattle cases and one goat case, no evidence of placental pathology was observed. In *N. caninum*-associated abortion, placental lesions are reported to be limited in nature, with focal areas of necrosis and non-suppurative inflammation confined to the cotyledons (Dubey *et al.*, 2006). Histopathology of foetal tissues is more typically used to confirm a diagnosis of *N. caninum* but unfortunately, foetal tissues were rarely available for examination in our study. However, the presence of placental lesions is not a pre-requisite for the diagnosis of *N. caninum*-associated abortion (Dubey & Schares, 2006), and the presence of *N. caninum* nucleic acids accompanied by evidence of dam seropositivity or seroconversion (as noted in all the positive abortion events included in this study component) remains supportive of a diagnosis of *N. caninum* in most cases.

Notably, evidence of co-infections with other pathogens was observed in three *N. caninum*-positive cases. One cattle case (SEBI-184), which met study case definitions for both *N. caninum* and BoHV-1, had evidence of placentitis accompanied by necrotising vasculitis. *N. caninum* and *C. burnetii* were detected concurrently in one cattle (SEBI-055) and one goat (SEBI-168) case respectively, although neither cases met the study case definition for *C. burnetii* due to insufficient bacterial load in placental or swab samples. It is therefore feasible that the presence of other bacterial and viral pathogens may have contributed to the severity of pathology observed in these abortions. Dubey et al (2006) note that multiple factors may interact to influence the severity of foetal or placental damage in *N. caninum*-associated abortions and therefore, it remains a plausible possibility that multiple infectious agents may have contributed to the foetal and placental damage that led to negative pregnancy outcome in these cases.

RVFV-associated abortions*:* Placental pathology was not detected in any of the six cattle abortion events that met the study case definitions for RVFV. Recent studies of RVFV-associated abortions in sheep have indicated that abortion results from severe

acute pathology of the placenta including haemorrhage within the placentomes of infected dams and necrosis of the foetal trophoblasts and maternal epithelial in the cotyledonary and intercotyledonary chorioallantois (Oymans *et al.*, 2020, Odendaal *et al.*, 2020). In a set of experimental RVFV infections in sheep, all abortions occurred within 7 days post infection indicating the acute nature of these placental changes, which were often observed in the absence of any notable foetal pathology (Oymans et al., 2020). Although cattle are reported to be slightly more resistant to RVFV-associated disease than sheep, the pathology associated with infection are thought to be consistent across these two species when abortions do occur (Schlafer & Foster, 2015).

Based on these recent reports, the absence of significant histopathologic findings in our samples may initially seem surprising. However, it is noteworthy that all of the RVFV-PCR positive placental samples that were examined in this study showed moderate to marked autolysis. Given the fact that necrosis is typically limited to the placental trophoblast layer in RVFV abortions, it seems plausible that histopathologic changes that are limited to superficial tissue layers may have been obscured by post-mortem degeneration. This finding highlights the importance of using adjunct diagnostic tests including RT-qPCR and serology to monitor for RVFV-associated abortions in livestock in areas with limited capacity for rapid sampling response and/or histopathologic examination.

BoHV-1-associated abortions: Two abortion events in cattle met our study case definitions for BoHV-1. In both of these cases, placentitis was observed. BoHV-1 abortion is associated with necrotic placentitis and vascular necrosis in experimental studies (Rodger *et al.*, 2007). As mentioned above, one animal, SEBI-184, that showed evidence of necrotising placentitis and vascular necrosis met both BoHV-1 and *N. caninum* case definitions and hence a co-infection cannot be ruled out. Similarly, invasive Gram-positive bacteria were seen in the placenta of the other BoHV-1 positive case suggesting that a bacterial aetiology, or co-infection, cannot be ruled out. It is also worth noting that our confidence in our study case definition for BoHV-1 is lower than for some other agents (e.g. *N. caninum*) as we relied only on evidence of seroconversion rather than direct demonstration of the pathogen.

However, when taken together, our serology and histopathology results indicate that BoHV-1 is likely to be a contributing cause to these abortions.

BVDV: Placental pathology was not detected in the one cattle abortion event associated with BVDV in our study. This is typical of BVDV infection in cattle (Murray, 1991, Schlafer & Foster, 2015). Foetal loss and abortion associated with BVDV occurs due to foetal infection with the pathogen. However, the outcome of these foetal infection depends upon both the timing of infection and the viral subtype (e.g., cytopathic or non-cytopathic viral biotypes). For example; infection during the first trimester (up to 80 days) can result in the birth of a persistently infection calf, whereas infection between 80-150 days gestation can lead to teratogenic effects in the foetus, which in turn may result in foetal death (Lanyon *et al.*, 2014, Schlafer & Foster, 2015). Unfortunately, due to the lack of foetal tissue samples available for histopathology, we were unable to evaluate foetal pathology in this case. However, the absence of placental pathology in this BVDV-positive case is consistent with our understanding of the pathogenesis of BVDV infection in pregnant cattle.

***Coxiella burnetii*:** Histopathology and IHC findings found evidence of placentitis with positive tissue labelling by IHC in one goat abortion event (SEBI-054) that met our case definition for *C. burnetii*-associated abortion. In this case, a very high bacterial load was present in the placental tissue as indicated by the low average Ct value of 12.5 obtained with the *IS1111*a qPCR assay. Acute, diffuse, suppurative placentitis accompanied by extensive necrosis is a typical finding in *C. burnetii*-associated abortion (Schlafer & Foster, 2015) and the presence of positive IHC antibody labelling within trophoblast cells further supports the causal relationship in this case.

Positive *IS1111*a qPCR results were obtained in other six abortion events with placental material for histopathology but no other cases met the Ct cut-off based on the minimum bacterial load recommended by OIE and EFSA for diagnosing *C. burnetii*-associated abortion (see supplementary material D) (Sidi-Boumedine *et al.*, 2010, World Organisation for Animal Health (OIE), 2018). No evidence of placental pathology or substantial amounts positive tissue labelling was detected in these cases either, which supports our use of the average placental Ct cut-off ≤ 32 for attributing clinical significance to and diagnosing abortions associated with *C. burnetii* in this

study. Placental tissue from one cattle abortion event (SEBI-055) did show a very small amount of granular tissue labelling, and this case also had an average placental Ct value of 32.2, very close to our diagnostic cut-off value of ≤32. This particular abortion event met study case definitions for *N. caninum* and due to the lack of placental pathology and the very low amount of IHC antibody labelling, this seems like a more likely aetiology for the observed abortion in this case. However, it remains a possibility that both pathogens were contributing to the aetiology of abortion in this case.

Other potential aetiologies of abortion detected by histopathology: Significant accumulations of bacteria were detected in two cattle cases (SEBI-103 and SEBI-172) that were negative by PCR or serology for all study pathogens. In both cases, moderate to high numbers of monomorphic coccobacilli were observed within the placental tissue or on the placental surface (see Table S1C). This suggests that other bacterial pathogens that were not included in our diagnostic strategy could also be making an important contribution to the problem of livestock abortion in Tanzania. Unfortunately, it was beyond the scope of this study to conduct further pathogen-specific diagnostic tests on these tissue samples, but this remains a priority question for future research in this sample set.

S1, C; Table C: Target pathogen serologic (ELISA) and molecular (PCR/RT-PCR) assay information, including primer/probe sequences and cycling conditions for samples from various livestock species.

**Pathogen**	**Livestock species tested**	**Serologic test**	**PCR type**	**Primer/ probe name**	**Primer/probe sequence (5' to 3') or commercial kit**	**Source**	**Cycling conditions (temperature/time)**					
							**RT step (1 cycle)**	**Initial heat inactivation (1 cycle)**	**Denaturation (35-45 cycles)**	**Annealing or Anneal/Extension (35-45 cycles)**	**Extension (35-45 cycles)**	**Final extension (1 cycle)**
Brucella spp.	C, G, S	Brucella COMPELISA Animal Plant Health Agency (APHA) UK	qPCR	IS7111 F	GGCCTACCGCTGCGAAT	(Matero et al., 2011)	n/a	95 °C/ 2 min	95 °C/ 5 sec	60 °C/ 30 sec	n/a	n/a
				IS711 R	TTGCGGACAGTCACCATAATG							
				IS711 probe	**FAM-**AAGCCAACACCCGGC-**MGBNFQ**							
				IPC	Taqman® Exogenous Internal Positive Control (Applied Biosystems)							
				bcsp31 F	GCTCGGTTGCCAATATCAATGC	(Probert *et al.*, 2004)	n/a	95 °C/ 2 min	95 °C/ 5 sec	60 °C/ 30 sec	n/a	n/a
				bcsp31 R	GGGTAAAGCGTCGCCAGAAG							
				bcsp31 probe	**FAM-**AAATCTTCCACCTTGCCCTTGCCATCA-**BHQ1**							
Chlamydia abortus	Serology: G, S only Molecular: C, G, S	In-house Moredun ELISA		CabMOMP F	GCGGCATTCAACCTCGTT	(Livingstone *et al.*, 2009)	n/a	95 °C/ 2 min	95 °C/ 5 sec	60 °C/ 5 sec	n/a	n/a
				CabMOMP R	CCTTGAGTGATGCCTACATTGG							
				CabMOMP probe	**FAM** – TGTTAAAGGATCCTCCATAGCAGCTGATCAG – **BHQ1**							
Coxiella burnetti	C, G, S	Thermofisher® PrioCHECK Ruminant Q Fever Ab		IS1111 F	CATCACATTGCCGCGTTTAC	(Roest *et al.*, 2011)	n/a	95 °C/ 2 min	95 °C/ 5 sec	60 °C/ 5 sec	n/a	n/a
				IS1111 R	GGTTGGTCCCTCGACAACAT							
				IS1111 probe	**FAM** – AATCCCCAACAACACCTCCTTATTCCCAC – **BHQ1**							

		Plate (ELISACO XLS5)										
Leptospira	Serology: C only Molecular: C, G, S	Linnodee® Bovine Leptospira Hardjo 5 Plate Solid ELISA kit (LBLK01)		lipL32 F	AAGCATTACCGCTTGTGGT G	(Stoddard *et al.*, 2009)	n/a	95 °C/ 2 min	95 °C/ 5 sec	60 °C/ 5 sec	n/a	n/a
				lipL32 R	GAACTCCCATTTCAGCGATT							
				lipL32 probe	FAM – AAAGCCAGGACAAGCGCCG – BHQ1							
Neospora and *Toxoplasma*	C, G, S	ID Screen® *Neospora caninum* Indirect ELISA<br><br>IDvet Toxoplasmosis Indirect Multi-species ELISA	1° and 2°Nested conventional PCR	NN1 F	TCAACCTTTGAATCCCAA	(Burrells *et al.*, 2013, Buxton *et al.*, 1998, Hurtado *et al.*, 2001)	n/a	95 °C/ 5 min	95 °C/ 1 min	55 °C/ 1 min	72 °C/ 1 min	72 °C/ 5 min
				NN1 R	CGAGCCAAGACATCCATT							
				NeoNP1 F	TACTACTCCCTGTGAGTTG							
				NeoNP1 R	TCTCTTCCCTCAAACGCT							
				ToxNP1 F	GTGATAGTATCGAAAGGTAT							
				ToxNP1 R	ACTCTCTCTCAAATGTTCCT							
BTV	C, G, S	n/a	RT-PCR	Commercial	Virotype BTV pan/8 RT-PCR Kit (VT280445) Indical®	n/a	50 °C/ 20 min	95 °C/ 15 min	95 °C/ 30 sec	57 °C/ 45 sec	68 °C/ 45 sec	n/a
Pestiviruses	Serology: C only Molecular: C, G, S	IDEXX® BVDV p80 Ab ELISA (P00645-5)		Commercial	Virotype BVDV RT-PCR Kit (VT280375) Indical®	n/a	50 °C/ 20 min	95 °C/ 15 min	95 °C/ 30 sec	57 °C/ 45 sec	68 °C/ 45 sec	n/a
RVF	C, G, S	ID Screen® Rift Valley Fever Competition Multispecies ELISA kit (RIFTC-10P)		RVS	AAAGGAACAATGGACTCTG GTCA	(Drosten *et al.*, 2002)	45 °C/ 10 min	95 °C/ 5 min	95 °C/ 5 sec	60 °C/ 30 sec	n/a	n/a
				RVAs	CACTTCTTACTACCATGTCC TCCAAT							
				RVP	**FAM** - AAAGCTTTGATATCTCTCAG TGCCCCAA -**BHQ1**							

Where: C = cattle, G = goats, S = Sheep

S1, D:

Genetic characterisation of *T. gondii* isolated from an abortion case

Methods: Any samples positive for *T. gondii* by ITS1 PCR were genotyped using PCR-RFLP targeting 10 genetic markers (SAG1, SAG2 (5'-3' SAG2 and alt.SAG2), SAG3, BTUB, GRA6, C22-8, C29-2, L358, PK1 and Apico). PCR-RFLP conditions for all markers were carried out as previously described (Hamilton *et al.*, 2015, Su *et al.*, 2010). Typing profiles were determined using RFLP banding profiles of reference strains RH (Type I), M4 (Type II variant; used for all markers except Apico where Type II strain Me49 was used instead) and NED (Type III).

Results: A single sheep sample was positive for *T. gondii* by PCR. Sequences was obtained for seven of 10 genetic markers. Although a complete genetic profile was not obtained, Type II alleles were identified at all seven amplified markers, indicative of a type II clonal genotype infection.

S1; E: Determining qPCR cut-off values for diagnosing *Coxiella burnetii* as the cause of abortion in ruminant livestock in Tanzania.

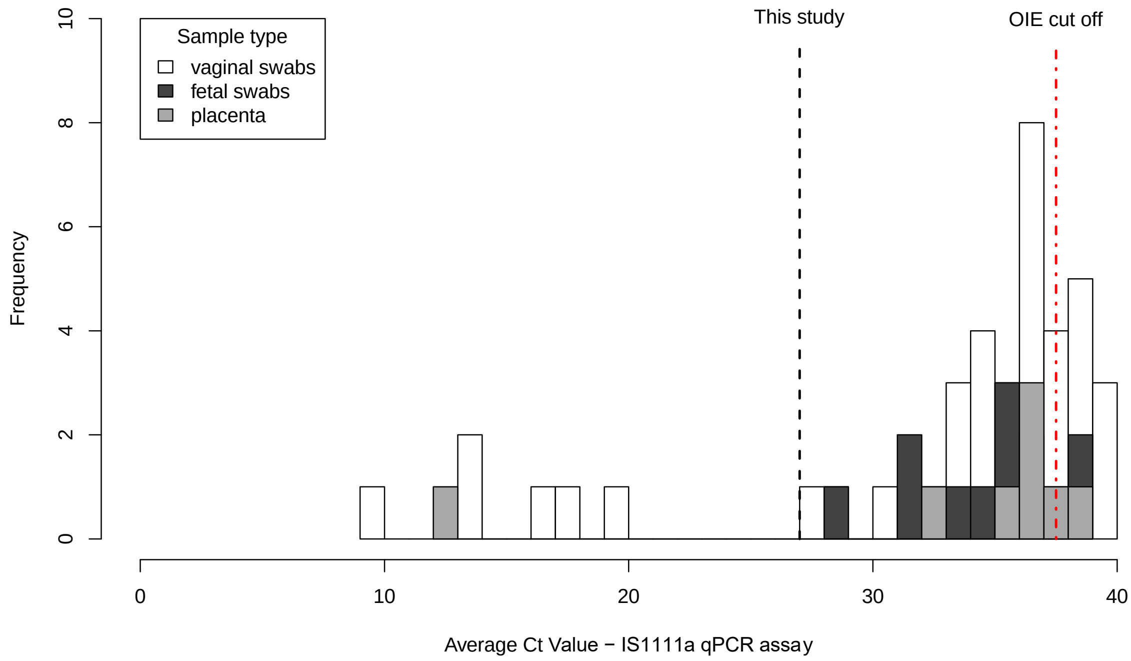

Figure S1; E: Histogram showing frequency distribution of average Ct values obtained from the IS1111a qPCR assay for the detection of *C. burnetii* in vaginal swab, fetal swab and placental tissue samples from ruminant livestock in Tanzania. The black dashed line indicates the cut-off Ct value (Ct value ≤ 27) selected for use in this study for attributing abortion events to *C. burnetii* infection. The red dash-dot line indicates the cut-off Ct value for vaginal swabs as stated by the OIE for attributing abortion events to *C. burnetii* infection (> 10^4 bacteria per swab, equivalent to a Ct value = 37.5 on our platform). The equivalent cut-off value for placental tissue quoted by the OIE is off the scale (> 10^4 bacteria per gram of placental tissue, equivalent to a Ct value = 42.7 on our platform). The histogram was plotted in R (R Core Team, 2016) using average Ct value data from all samples positive for *C. burnetii* by IS1111a qPCR assay (Klee *et al.*, 2006).

<u>Validation of selected Ct cut-off in placental tissue samples using with histopathology and immunohistochemistry results:</u> Histopathology and immunohistochemistry (IHC) for *C. burnetii* was performed on seven placental samples (five bovine, two caprine) that were qPCR-positive for *C. burnetii* by IS1111a qPCR testing (See Supplementary material Table

S1, B). Of these, one caprine placental sample had a Ct value ≤ 27 (average Ct value of 12.5) and all other samples had Ct values > 27 (range: 32.2 - 37.5). Placentitis with positive *C. burnetii* IHC labelling was detected in the caprine placental sample with a Ct ≤ 27. No evidence of significant placental pathology or significant IHC labelling was detected in any of the other samples. These findings support our selected Ct value cut-off to support diagnosis of *C. burnetii* associated abortions within our study population.

S1; F: SEBI-TZ Project Preliminary Laboratory Report Template

LABORATORY REPORT

"Zoonoses and Livestock Systems Research" (ZELS) Project
Supporting Evidence Based Interventions in Tanzania (SEBI-TZ)

University of Glasgow; Nelson Mandela African Institute of Science and Technology; Ministry of Livestock and Fisheries; Kilimanjaro Christian Medical Centre; Kilimanjaro Clinical Research Institute; Washington State University; University of Otago

---

The Executive Director
________ District Council
P.O. Box ___
(Ward)

Attention: (District Veterinary Officer)

Head of Household (to be completed by hand):…………………………………………

PRELIMINARY LABORATORY REPORT
Village:
Livestock Field Officer:
Sample collection date:
Date sample received:
Report date:
Field sample numbers: SEBI-___
Lab reference numbers:

Sample #	Sample Type/ Animal species	Tests performed	Result (DETECTED/ NOT DETECTED)
SEBI-TZ ___	___________	*Brucella* spp. PCR	_________
		Chlamydia spp. PCR	_________
		Coxiella burnetii PCR	_________
		Leptospira spp. PCR	_________
		Neospora PCR	_________
		Toxoplasma PCR	_________

Interpretation

Dr. Kate Thomas
SEBI-TZ
Kilimanjaro Clinical Research Institute
Zoonoses Laboratory Coordinator

For further consultation please contact Dr. Tito Kibona on [redacted]

Note: These tests were carried out according to internationally-recognised protocols. The KCRI Zoonoses Laboratory is not a reference laboratory.

C.C. Tanzania Veterinary Laboratory Agency, Arusha
Zonal Veterinary Investigation Centre, Arusha
Livestock Field Officer, _________

REFERENCES

Botta, C., G. Pellegrini, M. Hässig, T. Pesch, B. Prähauser, S. Wunderlin, F. Guscetti, M. Schneeberger, S. Schmitt, W. Basso, M. Hilbe, G. Schuler and N. Borel, 2019: Bovine fetal placenta during pregnancy and the postpartum period. *Vet Pathol,* **56,** 248-258.

Burrells, A., P. M. Bartley, I. A. Zimmer, S. Roy, A. C. Kitchener, A. Meredith, S. E. Wright, E. A. Innes and F. Katzer, 2013: Evidence of the three main clonal *Toxoplasma gondii* lineages from wild mammalian carnivores in the UK *Parasitol,* **140,** 1768-1776.

Buxton, D., S. W. Maley, S. Wright, K. M. Thomson, A. G. Rae and E. A. Innes, 1998: The pathogenesis of experimental neosporosis in pregnant sheep. *J Comp Pathol,* **118,** 267-279.

Drosten, C., S. Göttig, S. Schilling, M. Asper, M. Panning, H. Schmitz and S. Günther, 2002: Rapid detection and quantification of RNA of Ebola and Marburg viruses, Lassa virus, Crimean-Congo hemorrhagic fever virus, Rift Valley fever virus, dengue virus, and yellow fever virus by real-time reverse transcription-PCR. *J Clin Microbiol,* **40,** 2323-2330.

Dubey, J. P., D. Buxton and W. Wouda, 2006: Pathogenesis of bovine neosporosis. *J Comp Pathol,* **134,** 267-289.

Dubey, J. P. and G. Schares, 2006: Diagnosis of bovine neosporosis. *Veterinary Parasitol,* **140,** 1-34.

Hamilton, C. M., P. J. Kelly, P. M. Bartley, A. Burrells, A. Porco, D. Metzler, K. Crouch, J. K. Ketzis, E. A. Innes and F. Katzer, 2015: *Toxoplasma gondii* in livestock in St. Kitts and Nevis, West Indies. *Parasit Vectors,* **8,** 166.

Hurtado, A., G. Aduriz, B. Moreno, J. Barandika and A. L. García-Pérez, 2001: Single tube nested PCR for the detection of *Toxoplasma gondii* in fetal tissues from naturally aborted ewes. *Vet Parasitol,* **102,** 17-27.

Klee, S. R., J. Tyczka, H. Ellerbrok, T. Franz, S. Linke, G. Baljer and B. Appel, 2006: Highly sensitive real-time PCR for specific detection and quantification of *Coxiella burnetii*. *BMC Microbiol,* **6,** 2.

Lanyon, S. R., F. I. Hill, M. P. Reichel and J. Brownlie, 2014: Bovine viral diarrhoea: pathogenesis and diagnosis. *Vet J (London, England : 1997),* **199,** 201-209.

Livingstone, M., N. Wheelhouse, S. W. Maley and D. Longbottom, 2009: Molecular detection of *Chlamydophila abortus* in post-abortion sheep at oestrus and subsequent lambing. *Vet Microbiol,* **135,** 134-141.

Matero, P., H. Hemmilä, H. Tomaso, H. Piiparinen, K. Rantakokko-Jalava, L. Nuotio and S. Nikkari, 2011: Rapid field detection assays for *Bacillus anthracis*, *Brucella* spp., *Francisella tularensis* and *Yersinia pestis*. *Clin Microbiol Infect,* **17,** 34-43.

Murray, R. D., 1991: Lesions in aborted bovine fetuses and placenta associated with bovine viral diarrhoea virus infection. *Arch Virol Suppl,* **3,** 217-224.

Odendaal, L., S. J. Clift, G. T. Fosgate and A. S. Davis, 2020: Ovine Fetal and Placental Lesions and Cellular Tropism in Natural Rift Valley Fever Virus Infections. *Vet Pathol,* **57,** 791-806.

Oymans, J., P. J. Wichgers Schreur, L. van Keulen, J. Kant and J. Kortekaas, 2020: Rift Valley fever virus targets the maternal-foetal interface in ovine and human placentas. *PLOS Negl Trop Dis,* **14,** e0007898.

Probert, W. S., K. N. Schrader, N. Y. Khuong, S. L. Bystrom and M. H. Graves, 2004: Real-time multiplex PCR assay for detection of *Brucella* spp., *B. abortus*, and *B. melitensis*. *J Clin Microbiol,* **42,** 1290-1293.

R Core Team, 2016: R: a language and environment for statistical computing. *R Foundation for Statistical Computing, Vienna, Austria.* Available at: https://www.R-project.org/.

Rodger, S. M., J. Murray, C. Underwood and D. Buxton, 2007: Microscopical lesions and antigen distribution in bovine fetal tissues and placentae following experimental infection with bovine herpesvirus-1 during pregnancy. *J Comp Pathol,* **137,** 94-101.

Roest, H. I., R. C. Ruuls, J. J. Tilburg, M. H. Nabuurs-Franssen, C. H. Klaassen, P. Vellema, R. van den Brom, D. Dercksen, W. Wouda, M. A. Spierenburg, A. N. van der Spek, R. Buijs, A. G. de Boer, P. T. Willemsen and F. G. van Zijderveld, 2011: Molecular epidemiology of *Coxiella burnetii* from ruminants in Q fever outbreak, the Netherlands. *Emerg Infect Dis,* **17,** 668-675.

Schlafer, D. H. and R. A. Foster, 2015: Female Genital System. In: G. Maxie (ed.), *Jubb, Kennedy and Palmer's Pathology of Domestic Animals.* Elsevier Health Sciences.

Sidi-Boumedine, K., E. Rousset, K. Henning, M. Ziller, K. Niemczuck, H. I. J. Roest and R. Thiéry, 2010: Development of harmonised schemes for the monitoring and reporting of Q-fever in animals in the European Union. *European Scientific Report on Question No EFSA-Q-2009-00511.*

Stoddard, R. A., J. E. Gee, P. P. Wilkins, K. McCaustland and A. R. Hoffmaster, 2009: Detection of pathogenic *Leptospira* spp. through TaqMan polymerase chain reaction targeting the *LipL*32 gene. *Diag Microbiol Infect Dis,* **64,** 247-255.

Su, C., E. K. Shwab, P. Zhou and X. Q. Zhu, 2010: Moving towards an integrated approach to molecular detection and identification of *Toxoplasma gondii*. *Parasitol,* **137,** 1-11.

World Organisation for Animal Health (OIE), 2018: Q Fever. In: OIE (ed.), *OIE Terrestrial Manual*
